# Prediction of Blood Risk Score in Diabetes Using Deep Neural Networks

**DOI:** 10.3390/jcm12041695

**Published:** 2023-02-20

**Authors:** J. Quetzalcóatl Toledo-Marín, Taqdir Ali, Tibor van Rooij, Matthias Görges, Wyeth W. Wasserman

**Affiliations:** 1 Department of Anesthesiology, Pharmacology & Therapeutics, University of British Columbia, BC Children’s Hospital Research Institute, Vancouver, BC V5Z 4H4, Canada; 2 Department of Medical Genetics, University of British Columbia, BC Children’s Hospital Research Institute, Vancouver, BC V5Z 4H4, Canada; 3 Department of Computer Science, University of British Columbia, BC Children’s Hospital Research Institute, Vancouver, BC V5Z 4H4, Canada

**Keywords:** recurrent neural network, convolutional neural networks, deep learning, diabetes, continuous glucose monitor, blood glucose risk score, machine learning

## Abstract

Improving the prediction of blood glucose concentration may improve the quality of life of people living with type 1 diabetes by enabling them to better manage their care. Given the anticipated benefits of such a prediction, numerous methods have been proposed. Rather than attempting to predict glucose concentration, a deep learning framework for prediction is proposed in which prediction is performed using a scale for hypo- and hyper-glycemia risk. Using the blood glucose risk score formula proposed by Kovatchev et al., models with different architectures were trained, including, a recurrent neural network (RNN), a gated recurrent unit (GRU), a long short-term memory (LSTM) network, and an encoder-like convolutional neural network (CNN). The models were trained using the OpenAPS Data Commons data set, comprising 139 individuals, each with tens of thousands of continuous glucose monitor (CGM) data points. The training set was composed of 7% of the data set, while the remaining was used for testing. Performance comparisons between the different architectures are presented and discussed. To evaluate these predictions, performance results are compared with the last measurement (LM) prediction, through a sample-and-hold approach continuing the last known measurement forward. The results obtained are competitive when compared to other deep learning methods. A root mean squared error (RMSE) of 16 mg/dL, 24 mg/dL, and 37 mg/dL were obtained for CNN prediction horizons of 15, 30, and 60 min, respectively. However, no significant improvements were found for the deep learning models compared to LM prediction. Performance was found to be highly dependent on architecture and the prediction horizon. Lastly, a metric to assess model performance by weighing each prediction point error with the corresponding blood glucose risk score is proposed. Two main conclusions are drawn. Firstly, going forward, there is a need to benchmark model performance using LM prediction to enable the comparison between results obtained from different data sets. Secondly, model-agnostic data-driven deep learning models may only be meaningful when combined with mechanistic physiological models; here, it is argued that neural ordinary differential equations may combine the best of both approaches. These findings are based on the OpenAPS Data Commons data set and are to be validated in other independent data sets.

## 1. Introduction

In 2017, worldwide diabetes cases numbered 425 million, and it is estimated that by 2045 that number will increase to 700 million [1]. Diabetes can lead to a number of complications such as neuropathy, retinopathy, and nephropathy [2]. Many of the known risks associated with diabetes result from poor glucose management.

Both type 1 diabetes (T1D) and type 2 diabetes result from the interaction between the environment and the patient’s genes; however, their pathogenesis is distinct. T1D, the focus of this study, is due to the immune system destroying the beta-cells in the Islets of Langerhans—the cells responsible for insulin secretion and production. The resulting effect is currently treated with insulin to manage glucose. Current developments in the area of health-targeted wearable and implanted devices have leveraged glycemic management in several ways. First and foremost, the development of continuous glucose monitor (CGM) sensors has allowed for people living with T1D to access nearly real-time interstitial glucose values (reported every 1 to 5 min, depending on the kind of CGM sensor). This technology is replacing frequent finger-prick testing. In addition, through cloud services, patients can share their data with their care team and caregivers, catalyzing the generation of patient-health data which could be used for research. Furthermore, advances in insulin delivery technology have allowed for the use of closed-loop algorithms between the CGM sensor and insulin pumps, essentially acting as an artificial pancreas [3]. Despite advances in the use of health technology, poor glycemic management is still an issue. Patients still experience hypo- and hyper-glycemic events due to a range of interactions between stress, nutrition, exercise, medication administration, and other factors [4].

Using in silico and in vitro models has been pivotal to understanding the physiological mechanisms behind blood glucose dynamics. Ultimately, glucose variability is due to several complex processes involving but not limited to insulin, glucagon, epinephrine, cortisol, and growth hormones, which operate on different time scales. Mechanistic modeling has impacted research and therapy. In January 2008, the United States Food and Drug Administration (FDA) approved a simulator as a substitute for animal trials for the preclinical testing of control strategies in artificial pancreas studies. Furthermore, the simulator has been adopted by the Juvenile Diabetes Research Foundation (JDRF) Artificial Pancreas Consortium as a primary test bed for new closed-loop control algorithms [4,5,6].

To evaluate the degree of glucose mismanagement resulting in hypo- and/or hyperglycemia, multiple risk scores have been proposed. For instance, the coefficient of variability (CV) is defined as the ratio between the standard deviation and mean value of glucose in a sufficiently large time window, such that a high CV indicates high variability in glucose levels which contributes to sub-optimal glucose management. The variety and flavors of glucose risk scores are broad yet, currently, there is not a one-type-fits-all risk score nor has a consensus been reached for which risk score is best.

Despite ongoing research and funding directed toward diabetes treatment, which has resulted in clinical and technological progress, optimal glucose management remains an unsolved problem. In an effort to overcome these shortcomings, researchers have been designing and building deep learning models for glucose management and prediction [7,8,9,10,11,12] (see [13] for a review). Furthermore, competitions such as the Blood Glucose Level Prediction (BGLP) Challenge [14] have been instrumental in coordinating collective efforts and developing new deep-learning tools for glucose prediction, with the potential of improving the health and well-being of individuals with diabetes. While some results are promising, data-based approaches are still in the experimental phase. Most of the ongoing research is focused on glucose prediction in a time horizon between 5 and 180 min. As the prediction horizon increases, predictive accuracy typically decreases. Prediction in the time horizon beyond 150 min is highly desirable since meals, insulin injections, and external schedules operate on that time scale, whereas measurements within 150 min mainly correspond to the *pulsatile secretion of insulin* and *intrinsic oscillatory phenomena* regimes [15]. Felizardo et al. provide a thorough and detailed discussion of the current state of glycemia prediction [13]. They found that 80% of the reviewed literature corresponded to data-driven models, while the remaining pertained to hybrid models combining data-driven models with mechanistic modeling; 41% used only CGM data; 47% used CGM and insulin and/or meals data; and 12% used CGM, exercise and insulin and/or meals data. They report a broad spectrum of approaches and results, though an unsolved issue that stands out is that it is virtually impossible to compare performances between different methods due to both the variety of preprocessing techniques used and the application to different data sets from different populations. With regard to the latter, it has been shown that glycated hemoglobin (HbA1c) values, a key attribute for diabetes, can follow different paths in different populations [16]. Therefore, it is absolutely crucial to develop benchmarks that allow for the comparison between results obtained from different data sets.

In the present paper, a deep learning prediction framework is proposed whereby the prediction is conducted in a hypo- and hyperglycemia risk-score scale rather than a glucose concentration scale, leading to a more robust behavior during training. The risk score formula proposed by Kovatchev et al. [17] is used for this purpose, which symmetrizes the glucose data and properly weighs hyper- and hypoglycemic events. A metric to assess the model’s performance by weighing the prediction error with the blood glucose risk score is proposed and presented.

Several models with different typical architectures were trained, including a recurrent neural network (RNN), a gated recurrent unit (GRU), a long short-term memory (LSTM) network, and an encoder-like convolutional neural network (CNN). Historically, the first three types of architectures have been typically used for time series data, whereas convolutional neural networks have typically been used for image processing. A thorough comparison between different architectures is presented and discussed. The models were trained using the OpenAPS Data Commons [18] data set, comprising 139 individuals, each with tens of thousands continuous glucose monitor (CGM) data points. Root mean squared errors (RMSEs) of 16 mg/dL, 24 mg/dL, and 37 mg/dL were obtained for the CNN and for time horizons of 15, 30, and 60 min, respectively (throughout this paper we use mg/dL units for glucose unless stated otherwise). To evaluate the predictions, the performance results were compared with the *last measurement* (LM) prediction, through a sample-and-hold approach continuing the last known measurement forward. The obtained results are competitive when compared with other deep learning methods.

## 2. Materials and Methods

The OpenAPS Data Commons data set includes more than 184 individuals’ donated data, surpassing the number of days of data from other available data sets reported in the literature [18]. For this research and after following the OpenAPS Data Commons Research Guidelines [19,20], we were given access to *n* = 139 participants.

**Figure 1:**
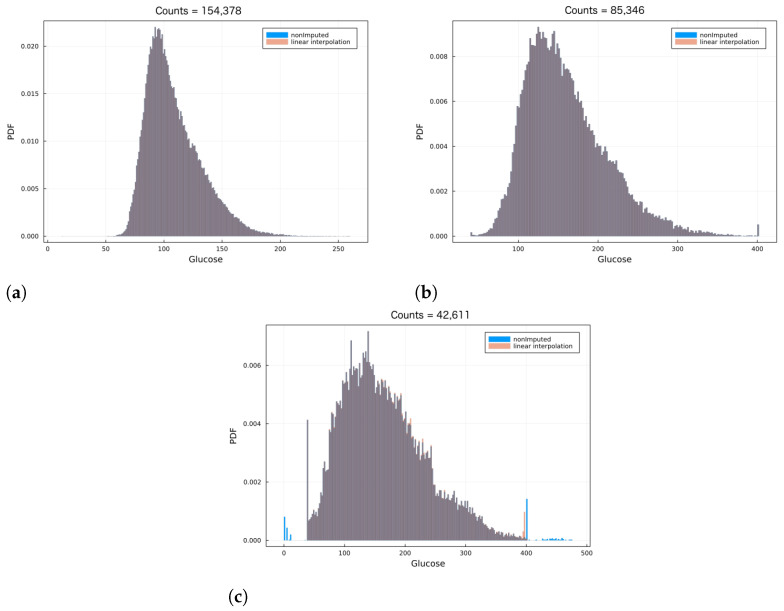
CGM normalized histograms before and after data imputation. Each panel corresponds to a different individual in the OpenAPS Data Commons. Each panel shows the CGM data before data imputation in blue, and after data imputation in red. The overlap between the two histograms in each panel suggests that the linear interpolation (proposed in Equation (2)) has little to no effect in changing the data structure. Notice the skewness in the histograms typical of CGM data. Panels (**a**), (**b**) and (**c**) correspond to 154,378, 85,346 and 46,611 glucose measurements, respectively.

The data used came from continuous glucose monitor (CGM) measurements. The time between two consecutive CGM measurements is typically five minutes. Before performing any analysis, the data were cleaned and, for consistency, we adhered to the same cleaning methodology as in [18]: all CGM entries different from a positive number (e.g., negative values, missing values, etc.) were removed; then, out-of-range CGM measurements (<39 or >400) were replaced with imputed values. Since the goal is to predict values in time series, data imputation was performed using linear interpolation. Denoting individual *p*’s CGM value to impute as $x_{t}^{p}$ at time *t*, provided $39\leq x_{t\pm \Delta t}^{p}\leq 400$; then, the linear interpolation yielded:

(1) $$x_{t}^{p}=\left\lfloor \frac{x_{t+\Delta t}^{p}+x_{t-\Delta t}^{p}}{2}\right\rfloor \;,$$

where ⌊•⌋ denotes the *floor* operator. In many cases where $39\leq x_{t-\Delta t}^{p}\leq 400$, $x_{t+\Delta t}^{p}\notin \left[39,400\right]$. Thus, generalizing Equation (1) leads to:

(2) $$x_{t+n\Delta t}^{p}=\left\lfloor \frac{x_{t+(n+1)\Delta t}^{p}+x_{t-\Delta t}^{p}}{2}\right\rfloor \;,$$

where *n* is a positive integer and $x_{t+n\Delta t}^{p}$ is the imputed value. Notice that the only constraint in order to apply Equation (2) is that the first and last measurements in the time series must be bounded between 39 and 400. Therefore, all initial and ending time series points were removed until the first and last points in each time series were in-bound, i.e., $39\leq x_{t_{initial}}^{p}\leq 400$ and $39\leq x_{t_{final}}^{p}\leq 400$, for all individuals. Afterwards, the linear interpolation in Equation (2) was applied to each of the individuals’ CGM data. Figure 1 shows the CGM data in a normalized histogram, for three different individuals. In blue corresponds to before imputing the data, while the red bins correspond to after data imputation via Equation (2). Notice the overwhelming overlap between non-imputed and imputed histograms (which yield a greyish-like color), suggesting the transformation due to data imputation had little to no effect in the data distribution. As part of the scope, the imputed data distribution should match that of the non-imputed data qualitatively, which is achieved using the previous simple and straightforward method. However, it is important to stress that there are a number of more sophisticated numerical methods (see for instance [21,22,23]). In the Appendix A we provide further insight regarding the imputed data; specifically, we show the percentage of imputed data per individual, as well as the mean and standard deviation of consecutive imputed values.

**Figure 2:**
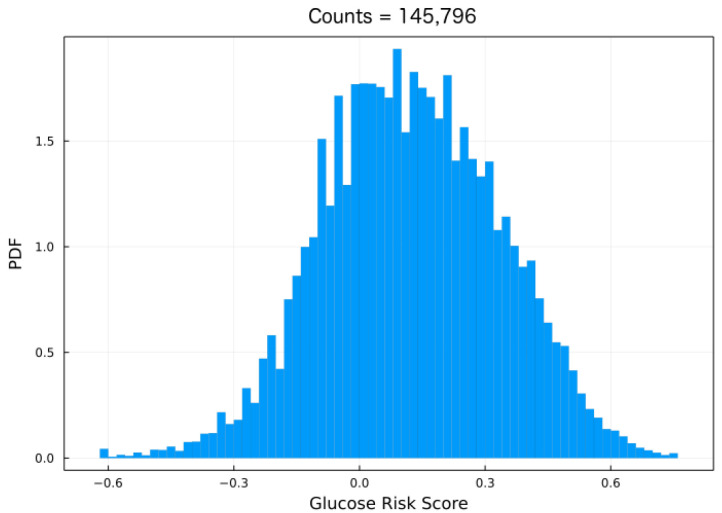
Normalized standardized variable histogram of an individual in the OpenAPS Data Commons data set. Equation (3) was used to map the CGM data into the BG-risk standardized variable. The histogram is more symmetrical than those shown in Figure 1.

After preprocessing the data, the CGM data were mapped into *risk score* data by using the symmetrization formulas proposed in [17,24], namely,

(3) $$y_{t}=\gamma \left(\ln^{\alpha }\left(x_{t}\right)-\beta \right)\;,$$

where α=1.084, β=5.381, and γ=1.509 are fitted parameters, and $y_{t}$ is the BG risk variable. Notice that the BG risk score function is defined as $BG\left(y_{t}\right)=10\cdot y_{t}^{2}$ [17]. As mentioned, the formula in (3) symmetrizes and rescales the glucose data such that $-\sqrt{1}0\leq y_{t}\leq \sqrt{1}0$. Most activation functions in neural networks (NNs) have a non-zero gradient in the interval $\left[-1,1\right]$ and, most importantly, zero gradients outside these bounds. For these reasons, the data is further transformed by dividing by $\sqrt{1}0$ such that $\xi_{t}=y_{t}/\sqrt{1}0$, where $\xi_{t}$ shall be referred to as the blood glucose (BG) risk-standardized variable or standardized variable. Figure 2 shows the resulting histogram in terms of the standardized variable ξ. The fact that the data are now bounded between −1 and 1 improves the model robustness as shown later.

In addition, symmetrizing the data also improves model performance by reducing the skewness, as seen in Figure 2. In a rather clever approach, in [25] the authors trained an LSTM to classify the risk by using the Kovatchev et al. risk score formula and considering 100 bins, yielding good performance. The approach in the present paper is similar although our method is strictly a regression model.

Different architectures were trained, namely, (1) an RNN, (2) a GRU, (3) an LSTM network, and (4) an encoder-like CNN. Models were built and trained in Julia using Flux [26]; the code used is available at [27]. To train the models, the length of the data sample was fixed to $10^{4}$ measurements per individual, corresponding to roughly 34 days. The individual with the largest coefficient variation was used together with nine randomly selected individuals as the training data set. Notice that the gradient of the mean absolute error (MAE) and the mean squared error (MSE) gradient are one and linear on the error, respectively. Therefore, if the prediction and ground truth values are between −1 and 1, the largest error per data point is 2; furthermore, most likely the error will be smaller than 1. Hence, the gradient using MSE is smaller than that using MAE. The loss function used was MAE when training models using the BG-risk standardized data which are bounded by −1 and 1, whereas for models trained using CGM data, having values between 39 and 400, the MSE loss function was used leading to a loss function gradient per data point proportional to the error. The length of each input sample was set to 10 points with an in-between gap equal to the prediction horizon for recurrent-like architectures, whereas for the CNN, input samples contained 16 previous consecutive points. It was found empirically that after 50 epochs, all models reached a plateau, while increasing the number of epochs beyond 50 epochs increased the test error leading to overfitting. Hence, all models were trained up to 50 epochs.

Multiple models were trained per architecture and per prediction horizon as described in Table 1. All architectures used hyperbolic tangent functions (*tanh*) as the activation functions, except for *RNN0* which used an *identity* activation function. All recurrent-like networks were composed of a single unit, whereas three convolutional layers and one fully connected layer were used for the CNN (Table 2).

Five replicas per architecture and per prediction horizon were trained as described in Table 1. All models were trained using the risk-score scale except for the RNN0 models, which were trained using data from the glucose concentration scale. All architectures were trained to predict either 15, 30, or 60 min into the future. The LSTM performance for the 15 min prediction horizon was significantly worse than the other architectures and is not reported here.

## 3. Results

This section presents results for the different architectures, namely the RNN, the GRU, the LSTM, and the CNN for three different prediction horizons: 15, 30, and 60 min (see Table 1). All replicas were evaluated on the test set consisting of the N−10=129 individuals’ data. For the time series of each individual, three metrics were computed, namely the ξ RMSE, the CGM RMSE (by inverting Equation (3)), and the CGM RMSE weighed with the normalized BG-risk score. For the latter, the normalized BG-risk score is defined as $BG\left(y_{t}\right)=y_{t}^{2}/10$, as mentioned previously.

**Figure 3:**
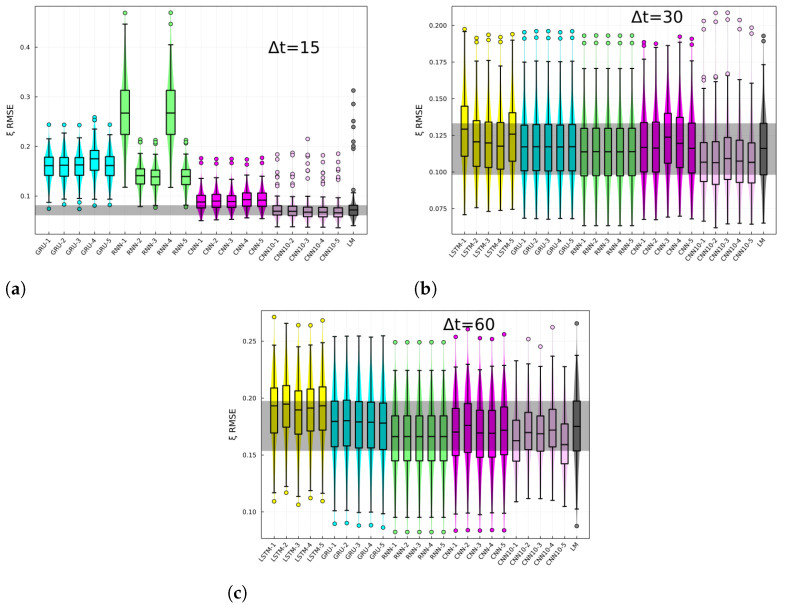
BG-risk standardized variable, ξ, RMSE for (**a**) 15, (**b**) 30, and (**c**) 60 min prediction horizon. Each panels shows the RMSE for different architectures specified in the *x*-axis and for the last-measurement prediction (LM). Each box corresponds to the model tested on N−10 individuals.

**Figure 4:**
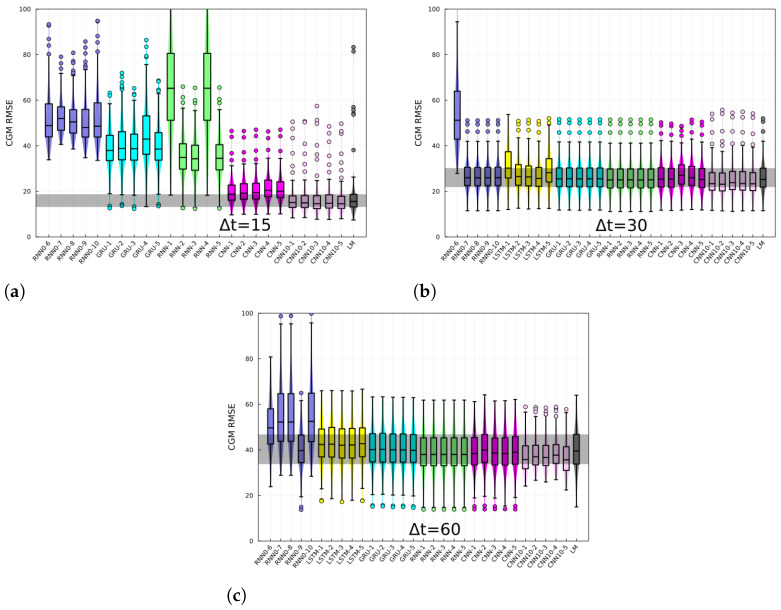
CGM prediction root mean squared error for (**a**) 15, (**b**) 30, and (**c**) 60 min horizons, and for different architectures. Each panels shows the RMSE for different architectures, specified in the *x*-axis and for the last-measurement prediction (LM). Each box corresponds to the model tested on N−10 individuals.

**Figure 5:**
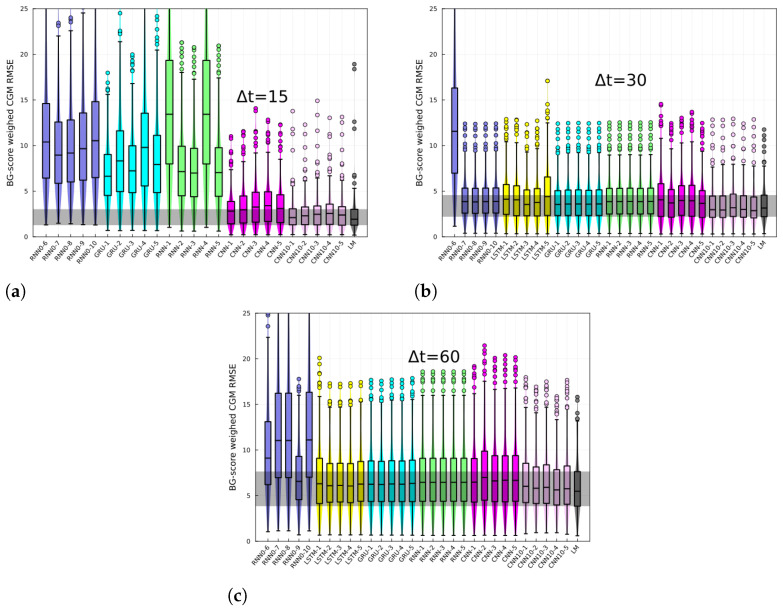
CGM prediction root mean squared error weighed with the normalized BG risk score for (**a**) 15, (**b**) 30, and (**c**) 60 min horizons, and for different architectures. Each panels shows the RMSE for different architectures, specified in the *x*-axis and for the last-measurement prediction (LM). Each box corresponds to the model tested on N−10 individuals.

The RMSE of the BG-risk standardized variable, ξ, histograms, and box plots are shown in Figure 3 for different prediction horizons. The different architectures are specified in the *x*-axis. The last-measurement prediction (LM), whereby the last known measurement is used as the prediction, is also included in the plot. The performance of recurrent-like architectures was highly variable for the 15 min prediction horizon, whereas the CNN consistently performed better than recurrent-like architectures. For 30- and 60-min prediction horizons, the performance is similar among different architectures. In Figure 4, the CGM RMSE is shown for different prediction horizons. The CGM prediction was obtained by inverting Equation (3), in order to convert BG-risk standardized data into CGM data. The CNN had the lowest RMSE for the CGM prediction, with values of 16 mg/dL, 24 mg/dL, and 37 mg/dL corresponding to prediction horizons of 15-, 30-, and 60-min, respectively. The performance of the replicas trained with CGM data (RNN0) fluctuated more than the RNN replicas, which suggests that using standardized data increases the training process’ robustness. In Figure 5, the CGM RMSE weighed by the BG-risk score is shown for different prediction horizons. Notice that there is no significant performance variation compared to Figure 4. However, the RMSE LM prediction slightly decreased relative to the rest of the NNs, although the decrease is not significant.

**Figure 6:**
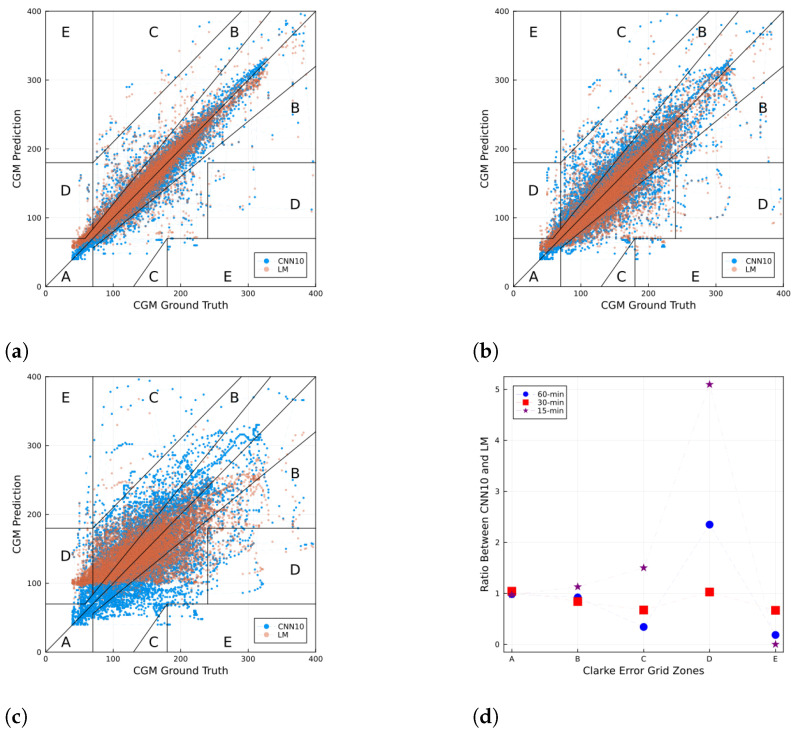
Clarke error grid for CGM prediction using the CNN10 and a sample in the test set for (**a**) 15, (**b**) 30, and (**c**) 60 min horizons. Panel (**d**) shows the ratio of the number of points between CNN10 and LM that fall in each zone in the Clarke error grid for the three different prediction horizons. For each zone in the *x*-axis, values above (below) 1 imply a larger (smaller) number of points from the CNN10 than the LM. In the case of the CNN10, the fraction of points in each zone are: (**a**) $\rho_{A}^{\left(15\right)}=0.908$, $\rho_{B}^{\left(15\right)}=0.068$, $\rho_{C}^{\left(15\right)}=0.003$, $\rho_{D}^{\left(15\right)}=0.021$, $\rho_{E}^{\left(15\right)}=0$; (**b**) $\rho_{A}^{\left(30\right)}=0.819$, $\rho_{B}^{\left(30\right)}=0.160$, $\rho_{C}^{\left(30\right)}=0.004$, $\rho_{D}^{\left(30\right)}=0.016$, $\rho_{E}^{\left(30\right)}=0.001$; and (**c**) $\rho_{A}^{\left(60\right)}=0.566$, $\rho_{B}^{\left(60\right)}=0.345$, $\rho_{C}^{\left(60\right)}=0.003$, $\rho_{D}^{\left(60\right)}=0.085$, $\rho_{E}^{\left(60\right)}=0.001$.

For a more detailed performance inspection, Figure 6 shows a Clarke error grid [28] for CGM prediction using the CNN10 and a sample in the test set for 15, 30, and 60-min horizons. The Clarke error grid shows the differences between a blood glucose predictive measurement and a reference measurement, and the clinical significance of the differences between these values. The *x*-axis corresponds to the ground truth, while the *y*-axis corresponds to the prediction. The diagonal line corresponds to when the prediction value is the same as the ground truth. This grid is split into five zones: Zone A is defined as clinical accuracy, zone B as clinically acceptable, whereas zones C, D, and E are considered clinical errors. Figure 6d shows the ratio of the number of points between CNN10 and LM that fall in each zone in the Clarke error grid for the three different prediction horizons. For each zone in the *x*-axis, values above 1 imply a larger (smaller) number of points from the CNN10 than the LM. Conversely, values below 1 imply a smaller number of points from the CNN10 than the LM.

**Figure 7:**
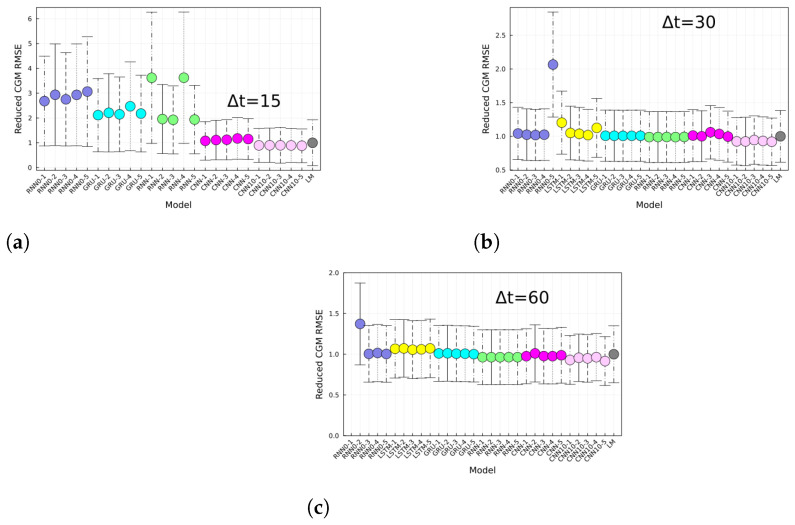
CGM prediction root mean squared error reduced by the LM prediction for (**a**) 15, (**b**) 30, and (**c**) 60 min horizons, and for different architectures. Each panels shows the RMSE for different architectures specified in the *x*-axis and for the last-measurement prediction (LM). The RMSE of the given model is divided by the RMSE of the LM. Models below (above) 1 perform better (worse) than the LM approach.

Lastly, shown in Figure 7 is the RMSE of the different replica models divided by the RMSE of the LM approach. The interpretation is straightforward, i.e., models below 1 perform better than the LM approach, and models above 1 perform worse. The average performance of CNN is slightly better than the LM approach; however, is not statistically significant.

## 4. Discussion

A total of three different metrics were shown for the different architectures, namely the RNN, the GRU, the LSTM, and the CNN and for three different prediction horizons: 15, 30, and 60 min. The performance of the models using BG-risk standardized data is consistent with that of CGM data, which supports the use of BG-risk standardized data for glucose prediction. Furthermore, in the case of RNNs, models trained using CGM data (models with prefix *RNN0*) performed significantly worse than those trained with BG-risk standardized data for the CGM 15 min prediction horizon. The latter exhibited more variability between replicas. In the case of 30 and 60 min prediction horizons, performances are similar although models trained using CGM exhibited more variability between different replicas. In the case of recurrent-like networks, no clear improvements were observed when comparing the different architectures described in Table 1.

The CNN architectures demonstrated the best performance in the 15 min prediction horizon. While the RNN and the GRU performed similarly, the GRU was more robust during training. The RNN0 performed significantly worse than the GRU and the RNN. The 15 min prediction horizon was significantly worse when using the LSTM compared to the rest of the architectures and is not reported here. For 30 and 60 min prediction horizons, the results are consistent throughout replicas although no significant improvement in performance compared to other architectures was obtained. It is worth noting that in the 60 min prediction horizon, the lower bound CGM RMSE is in the range of ≤20 for all models except RNN0 and CNN10. However, it is important to highlight that the CNN10 performance is able to generalize better than the rest by reducing outliers. This is a rather interesting effect, since by reducing the outliers, there are individuals for which models other than the CNN10 yield a lower CGM RMSE and therefore a better prediction. Had we trained our models per individual data set using a smaller data set, we most likely would have interpreted the lower bound outlier performance as a benchmark which may have led to a misinterpreted good performance.

Finally, no model displayed a significant improvement in performance compared to the LM prediction. This suggests that the trained networks were mainly memorizing the last known measurement. This behavior has also been seen in other work [12], where the prediction lags the ground truth. Furthermore, in [29] the authors propose using LSTM NNs to predict future cryptocurrency prices and also observed a lag between the ground truth value and the prediction, suggesting the lag is not a feature of the particular time series used. Therefore, computing the relative model performance, defined as the performance ratio between deep learning model and the LM prediction, as shown in Figure 7, can lead to a better benchmark. Another way to visualize performance in a finer scale is by using a Clarke error grid as shown in Figure 6. It is worth noting from the ratio of the number of points per zone obtained from the CNN10 model and the LM approach (see Figure 6d) that zones A and B contain approximately the same number of points from both methods, whereas for zones C, E, and D the ratios go over and under 1, further showing that the NN is performing similar to the LM approach.

Typical CGM data sets are obtained from individuals wearing CGM sensors. As the sensors’ output can inform their actions, there is the potential for later measurements to be influenced by earlier measurements within a feedback loop. However, such decisions are not well captured in the data sets. For instance, when an individual’s glucose goes above a given threshold, the individual receives a notification, and the individual may or may not act upon it (e.g., via an insulin dose). The individual’s action is not captured in the CGM data set though the effect of the action is. Therefore, a neural network may learn the effect (lowering glucose) but not the cause (the individual’s action). The same argument holds for the scenario where glucose goes below a given threshold, which is medically more concerning for the individual. Therefore, the CGM data set is biased by default; hence, even CGM data-driven models with perfect prediction cannot be used as stand-alone CGM management protocols. Instead, they can be used as an extra layer for glucose management. One may be compelled to think that having contextual information on the action taken by the patient (e.g., insulin data) can circumvent this shortcoming in data-driven models; yet, due to the multiple physiological and activity-wise factors contributing to glucose behavior, reliable stand-alone data-driven models would require multiple sources of data capturing all these contributing factors, beyond CGM and insulin data. This fundamental limitation is inherent in data-driven modeling. Conversely, in silico models have successfully understood and managed glucose behavior [3,4,5,6]. Embedding neural networks as mathematical operators in ordinary differential equations (NODEs) is a growing field, which is beginning to yield promising results ranging from OpenAI’s Dalle 2 [30] to immune system digital twin modeling [31].

## 5. Conclusions

In this paper, a deep learning prediction framework was proposed and tested. The prediction is made using a hypo- and hyperglycemia risk-score scale rather than attempting to predict glucose concentrations, leading to more robust training. The risk score formula proposed by Kovatchev et al. [17] was used for this purpose, which symmetrizes the glucose data. Several models with different architectures were trained and tested. No significant improvement was found from using deep learning models compared to LM prediction, suggesting that the trained networks were potentially also memorizing the last known measurement. The performance is highly dependent on architecture and the prediction horizon. We presented three metrics for evaluating the performance of our predictive model for continuous glucose monitoring (CGM) data: the standardized risk-score root mean squared error (RMSE), the CGM RMSE, and the CGM RMSE weighted by the normalized BG-risk score. The latter metric considers the associated risk of mis-prediction and could have the potential for use as a clinical marker. However, further investigation is required. We emphasize that our results should be interpreted relative to the data set used and thus have presented the ratio of the prediction error to the last-measurement error for this purpose.

Finally, it is important to note that our method trains the model using 1 or 10 data sets and tests it on the remaining 129 data sets, as this allows us to provide statistics on the model’s performance.

Two main conclusions were drawn/firstly, the need to benchmark model performance using LM prediction to normalize benchmark results obtained from different data sets and secondly, that model agnostic data-driven deep learning models may only be meaningful when combined with mechanistic physiological models.

More diverse data sets are needed to enable better data-driven models trained on a composite data set. Further emphasis on hybrid models is key for better glucose management.

## Figures and Tables

**Table 1:** Model prefix and the corresponding description. Several replicas per model per prediction horizon (PH) were trained. Models were named as *Model prefix*_*number*. The model prefix defines the architecture, the training set, and the training scale used.

Model Prefix	Replicas	PH	Description
RNN0	5	15/30/60	One individual’s data in glucose scale
RNN	5	15/30/60	One individual’s data in risk-score scale
GRU	5	15/30/60	One individual’s data in risk-score scale
LSTM	5	30/60	One individual’s data in risk-score scale
CNN	5	15/30/60	One individual’s data in risk-score scale
CNN10	5	15/30/60	Ten individuals’ data in risk-score scale

**Table 2:** CNN architecture. The input consisted of the 16 previous measurements arranged in a 4 × 4 lattice with one channel. Regularizers were not required due to the kernel sizes and the NN size overall.

Type of Layer	Kernel	Padding	Stride	Output Channels	Activation Function
Convolution	2 × 2	0	1	4	tanh
Convolution	2 × 2	0	1	8	tanh
Convolution	2 × 2	0	1	16	tanh
Fully Connected	-	-	-	1	tanh

**Figure A1:**
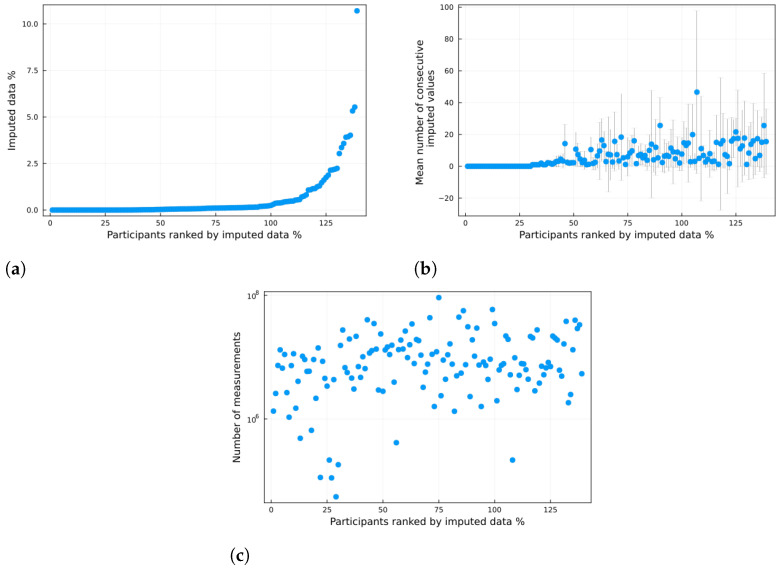
(**a**) Imputed data percentage per individual. (**b**) Mean number of consecutive imputed values per individual. The error bars correspond to the standard deviation. (**c**) Number of measurements per individual. In all plots, the *x*-axis is the ranked imputed data percentage from smallest to largest.

## Data Availability

The data is part of the OpenAPS Data Commons [18]. For access, see the Research Guidelines [20] and fill the form found therein. The tools presented in the methods section can be found in [27].

## Appendix A

In Figure A1, we show the percentage of imputed data per individual (panel a), the mean and standard deviation of consecutive imputed values per individual (panel b), and the number of measurements per individuals (panel c). In all plots, we have arranged the data by ranking the imputed data percentage per individual from smallest to largest.
